# Community Aftercare Linkage and Acute Clinical Outcomes Among Patients Presenting to the Emergency Department Following Suicide Attempts: A Retrospective Cohort Study

**DOI:** 10.3390/healthcare14213712

**Published:** 2026-11-02

**Authors:** Ji Hong Kim, Suk-Young Kim, Jisu Kim, Jae Hun Oh, Jong Ho Zhu, Mi Kyong Kwon, Seung Pill Choi, Ju Yeon Cho, Dabeen Han, Young Min Ha, Moon Sun Jin, Soo Hyun Kim

**Affiliations:** 1 Department of Emergency Medicine, Eunpyeong Saint Mary’s Hospital, College of Medicine, The Catholic University of Korea, Seoul 03312, Republic of Koreakimjisu@cmcnu.or.kr (J.K.); emojh@catholic.ac.kr (J.H.O.); emvic98@catholic.ac.kr (S.P.C.); 2 Department of Psychiatry, Eunpyeong Saint Mary’s Hospital, College of Medicine, The Catholic University of Korea, Seoul 03312, Republic of Korea; ksyh89@catholic.ac.kr; 3 Crisis Intervention Center for Life, Social Work Team, Eunpyeong Saint Mary’s Hospital, Seoul 03312, Republic of Korea

**Keywords:** suicide attempt, emergency department, community aftercare, continuity of care, Risk–Rescue Rating Scale, case management

## Abstract

**Highlights:**

**What are the main findings?**

- Among 975 discharge survivors, 530 (54.4%) accepted hospital-based case management and 213 (21.8%) achieved successful community aftercare linkage; 40.2% of accepting survivors were linked.
- College attendance, psychiatric history, and previous suicide attempts were associated with successful linkage.

**What are the implications of the main findings?**

• Initial registration is a measurable transition milestone, not evidence of sustained mental health treatment.
• The findings support further evaluation of the hospital-to-community pathway rather than causal or practice-changing conclusions.

**Abstract:**

Background/Objectives: This study examined factors associated with successful community aftercare linkage after emergency department (ED) presentation following suicide attempts, with acute hospital outcomes evaluated separately. Methods: This single-center retrospective cohort included 1035 patients aged ≥ 15 years presenting during 2023–2025. Successful linkage was defined as confirmed initial registration at a community mental health center. Multivariable logistic regression assessed linkage among 975 discharge survivors; intensive care unit (ICU) admission and in-hospital mortality were secondary outcomes in the full cohort. Results: Among survivors, 530 (54.4%) accepted hospital-based case management and 213 (21.8%) achieved linkage, representing 40.2% of accepting survivors. College attendance (adjusted odds ratio [aOR], 1.69; 95% confidence interval [CI], 1.12–2.55), psychiatric history (aOR, 1.69; 95% CI, 1.13–2.55), and previous suicide attempts (aOR, 1.81; 95% CI, 1.27–2.56) were associated with linkage. The intubation estimate was uncertain (aOR, 0.52; 95% CI, 0.26–1.06). Model discrimination was modest (area under the receiver operating characteristic curve, 0.710), with evidence of lack of fit (Hosmer–Lemeshow *p* = 0.009). ICU admission occurred in 218 patients (21.1%) and in-hospital death in 60 (5.8%). Conclusions: Initial acceptance and completed linkage were distinct stages of this care pathway. The associated factors may inform further investigation of care transitions, but initial registration does not establish sustained treatment engagement.

## 1. Introduction

Suicide prevention requires attention both to acute survival and to subsequent care [1]. Patients who survive a suicide attempt remain at risk of later suicidal behavior and suicide [2,3]. The period after discharge is an important opportunity for continued assessment and support [4,5]. For emergency departments (EDs), this creates a practical challenge: medical stabilization must be accompanied by arrangements for appropriate follow-up, without assuming that discharge or agreement to receive support ensures continuity of care.

Care-transition guidance recommends coordinated follow-up after an acute suicidal crisis [6,7]. Reviews and trials of brief contact and other post-attempt interventions have evaluated subsequent suicidal behavior [8,9,10], including ED-SAFE, the Attempted Suicide Short Intervention Program (ASSIP), and caring text messages [11,12,13]. A meta-analysis by Doupnik et al. found that brief acute-care suicide prevention interventions were associated with improved linkage to follow-up care and fewer subsequent attempts [14]. However, the outcomes of these interventions are not interchangeable with initial registration in a particular community service.

Research on psychiatric discharge highlights the organizational complexity of hospital-to-community transitions [15], while longitudinal evidence in severe mental illness links continuity of care with health outcomes [16]. Qualitative studies following self-harm emphasize patients’ concerns about assessment and the influence of care experiences on subsequent help-seeking [17,18]. In Korea, population-based evidence also documents stigma toward suicide attempts and depression [19]. These findings provide context for examining access to services, although stigma, health literacy, and patient experience cannot be inferred from routine registration records alone.

Previous studies have examined acceptance of guidance, community aftercare, registration, and loss to follow-up [20,21,22,23]. These represent different stages of care. Non-registration may limit access to services delivered through a specific referral pathway, although patients may receive mental health care elsewhere. Clarifying the factors associated with completed initial linkage is therefore useful without treating non-linkage as absence of all psychiatric care.

Medical severity remains relevant to the acute encounter. The Risk–Rescue Rating Scale (RRRS) characterizes risk and rescue circumstances, and recent studies have revisited its measurement properties and contribution to lethality assessment [24,25,26]. Other ED research has examined factors associated with serious suicide attempts [27]. We primarily evaluated factors associated with successful community aftercare linkage among discharge survivors. Separate secondary analyses examined intensive care unit (ICU) admission, in-hospital mortality, and recorded RRRS measures within the same cohort; these endpoints were not treated as directly comparable or competing outcomes.

## 2. Materials and Methods

### 2.1. Study Design and Setting

This retrospective observational cohort study was conducted at Eunpyeong St. Mary’s Hospital, an 830-bed teaching hospital in Seoul, Republic of Korea, with approximately 50,000 annual ED visits. The institutional review board approved the study (PC26RISI0097; 30 April 2026) and waived informed consent because of its retrospective design.

### 2.2. Study Population

**Figure 1:**
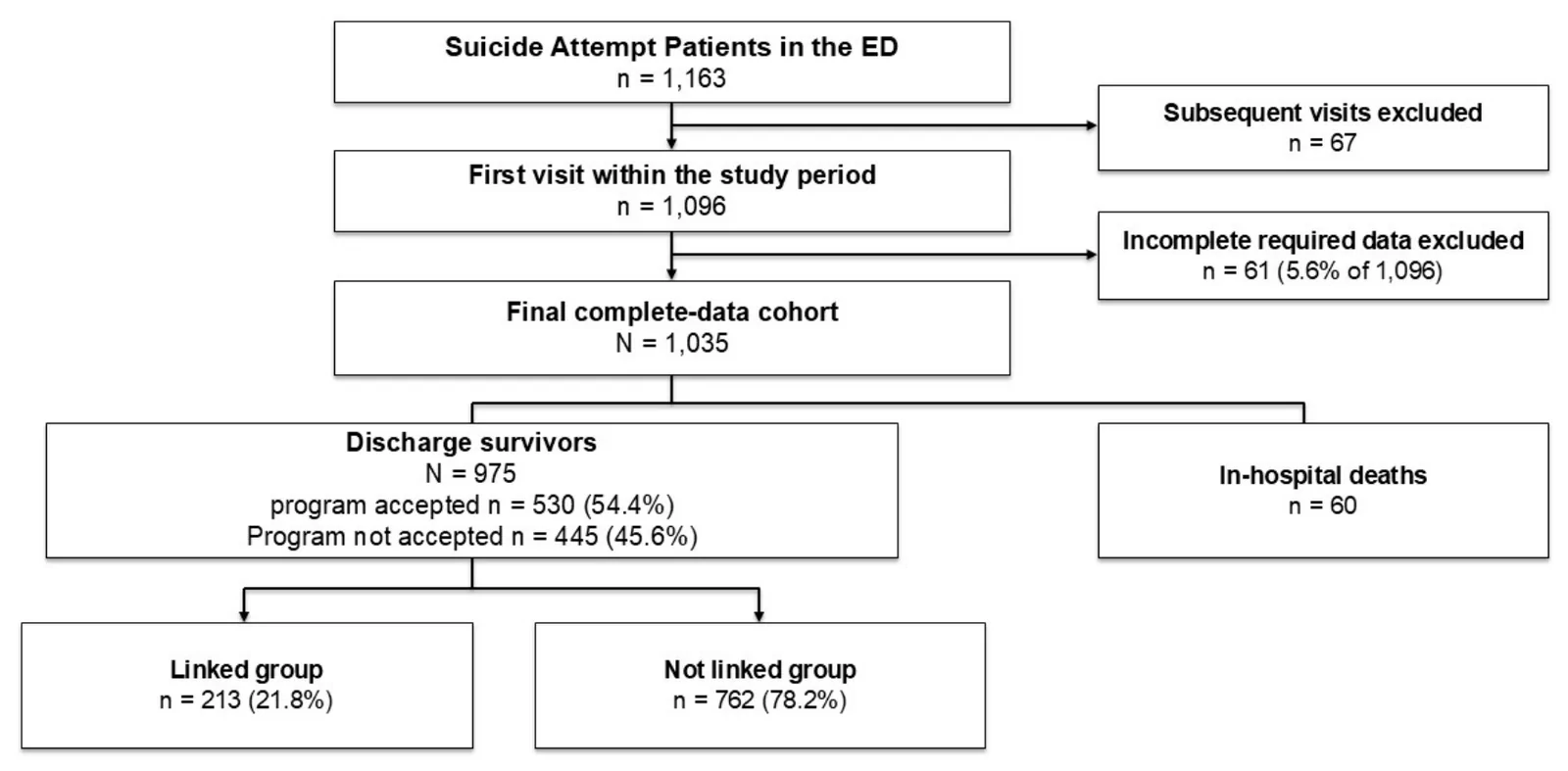
Patient selection and successful community aftercare linkage. Percentages in the primary cohort use 975 discharge survivors unless another denominator is specified. All 213 linked patients were among the 530 accepting survivors; the remaining 317 accepting survivors and 445 nonacceptors comprised the group classified as not linked. Initial linkage was confirmed by community-center registration; subsequent retention was not part of the outcome.

Patients aged ≥ 15 years presenting between January 2023 and December 2025 were identified from ED records and the National Emergency Department Information System (NEDIS) category used locally to report suicide attempts/intentional self-harm. Suicide-attempt status followed clinical reporting rather than a separate research adjudication of suicidal intent. Of 1163 visits, 67 subsequent visits were excluded by retaining only each patient’s first presentation within the study period, leaving 1096 unique patients. After review of initial and subsequent clinical and case-management records, 61 patients (5.6%) with incomplete required data were excluded, counting each patient once regardless of the number of missing variables. The final complete-case cohort comprised 1035 patients (Figure 1). The 975 discharge survivors formed the primary linkage cohort; all 1035 patients contributed to the secondary acute-outcome analyses.

The sample size was determined by the fixed retrospective study period; no a priori sample-size calculation was performed. The primary model included 12 covariate coefficients for 213 linkage events (17.8 events per coefficient, excluding the intercept).

### 2.3. Post-Attempt Management Program

Patients were offered hospital-based case management during admission or after discharge. Service acceptance was defined as agreement to participate documented by the case-management team. The program comprised four routine contacts, with up to two additional contacts according to need, to assess psychological status, support continued psychiatric care, and arrange referral to a regional community mental health center. Referral followed completion of hospital-based management. Patients discharged against medical advice (DAMA) were not excluded because of that disposition and were also offered the program and contacted for follow-up. Program completion and referral counts were not separately available for this analysis.

### 2.4. Variables and Measurements

Demographic, social, clinical, and treatment variables were abstracted retrospectively from ED records, subsequent clinical assessments, and case-management documentation. Psychiatric history included a documented diagnosis or treatment history, including discontinued treatment. College attendance indicated entry into college or university regardless of graduation; religious affiliation indicated any recorded religion. Employment included students, consistent with the original coding. Previous suicide attempt referred to events before the index visit. Reported reasons for the index attempt were based on patient or caregiver statements; ‘unknown’ was a recorded category, not an imputed absence of a reason. Altered mental status was defined as an initial Glasgow Coma Scale (GCS) score ≤ 14.

RRRS component responses were obtained from clinical documentation, including structured poisoning templates with predefined categorical responses completed by the initial physician and supplemented by later interviews. Recorded scores were extracted or calculated from the same predefined item responses. The dataset’s RRRS summary score was the sum of risk and rescue component scores; the risk–rescue ratio was 100 × risk/(risk + rescue). Intubation, vasopressor use, psychiatric consultation, and financial support were recorded as present or absent. Refusal/non-registration circumstances were obtained from case-management records or center feedback; the coded refusal-reason categories were available only for program nonacceptors and were analyzed accordingly.

### 2.5. Outcomes

Successful community aftercare linkage was defined as confirmed initial registration at the receiving community mental health center. Referral outcomes were verified by faxed feedback documenting registration, non-registration, or refusal. Program nonacceptors and patients with documented non-registration were classified as not linked; unresolved outcomes were treated as missing and excluded, not assumed to represent non-linkage. Outcome confirmation for late-2025 presentations continued into 2026. Linkage was assessed as an ever-confirmed program outcome rather than within a uniform post-discharge interval. Subsequent registration retention was reviewed at quarterly case meetings but was not part of the primary outcome.

Secondary outcomes were ICU admission during the index episode and death before hospital discharge. Later visits to this ED were not counted as new cohort members. Post-discharge re-attempt was not analyzed as an outcome because attempts treated elsewhere could not be systematically captured.

### 2.6. Statistical Analysis

Continuous variables were summarized as medians (interquartile ranges [IQRs]) and categorical variables as counts (percentages). Unadjusted comparisons used the Mann–Whitney U test, Pearson chi-square test, or Fisher exact test for sparse tables; larger sparse tables used Monte Carlo estimation with 200,000 replicates. Linkage covariates were selected on clinical relevance or univariable *p* < 0.10 in the initial analysis. The resulting 12 covariates were entered simultaneously and retained unchanged in the survivor-restricted model and full-cohort sensitivity analysis. The seven-covariate exploratory acute-outcome models included age, psychiatric history, previous suicide attempt, RRRS summary score, altered mental status, intubation, and vasopressor use to represent age, prior psychiatric/suicidal history, and clinical severity.

Conventional logistic regression provided adjusted odds ratios (aORs), Wald 95% confidence intervals (CIs), and two-sided *p*-values for linkage and ICU admission. Quasi-complete separation in the mortality model prompted Firth penalized logistic regression, with profile penalized-likelihood CIs and likelihood-ratio *p*-values. Variance inflation factors (VIFs) assessed collinearity. Apparent discrimination was evaluated by the area under the receiver operating characteristic curve (AUC; DeLong CI); model calibration was assessed using the Hosmer–Lemeshow goodness-of-fit test. No multiplicity adjustment was applied to the reported exploratory comparisons. Analyses used IBM SPSS Statistics, version 22.0 (IBM Corp., Armonk, NY, USA) and R, version 4.5.3 (R Foundation for Statistical Computing, Vienna, Austria).

## 3. Results

### 3.1. Baseline Characteristics

The final cohort included 1035 patients, with a median age of 34 years (IQR, 23–54); 330 (31.9%) were male. Poisoning accounted for 963 presentations (93.0%), drowning/asphyxiation for 58 (5.6%), and fall/firearm-related injury for 14 (1.4%). Psychiatric history was recorded in 634 patients (61.3%) and previous suicide attempts in 322 (31.1%). Altered mental status occurred in 360 patients (34.8%), intubation in 123 (11.9%), and vasopressor use in 97 (9.4%). Table 1 summarizes the cohort.

### 3.2. Service Acceptance and Community Aftercare Linkage

In the full cohort, 588/1035 patients (56.8%) had documented service acceptance; 58 of these patients died in hospital. Among the 975 discharge survivors, 530 (54.4%) accepted the program and 213 (21.8%) achieved successful community aftercare linkage. All linked patients had accepted the program, corresponding to 213/530 (40.2%) of accepting survivors. The group classified as not linked comprised 317 accepting survivors and 445 nonacceptors (total, 762). These counts distinguish acceptance from completed linkage but do not quantify attrition at individual intermediate stages.

Among the 445 survivor nonacceptors, documented refusal categories were psychological burden in 192 (43.1%), physical/practical limitations in 156 (35.1%), and low insight or perceived need in 97 (21.8%). These categories describe program nonacceptance, not reasons for failed linkage after acceptance. Among survivors, psychiatric consultation was recorded in 99/213 linked patients (46.5%) and 254/762 not linked patients (33.3%); financial support was recorded in 54 (25.4%) and 47 (6.2%), respectively (Table 2).

### 3.3. Factors Associated with Successful Community Aftercare Linkage

In the survivor-restricted model (Table 3), college attendance (aOR, 1.69; 95% CI, 1.12–2.55; *p* = 0.012), psychiatric history (aOR, 1.69; 95% CI, 1.13–2.55; *p* = 0.011), and previous suicide attempt (aOR, 1.81; 95% CI, 1.27–2.56; *p* < 0.001) were associated with higher odds of linkage. Employment had an aOR of 1.455 (95% CI, 0.999–2.120; *p* = 0.051). The intubation estimate was uncertain (aOR, 0.52; 95% CI, 0.26–1.06; *p* = 0.073). In the full-cohort sensitivity analysis, the intubation estimate was similar (aOR, 0.49; 95% CI, 0.24–0.99; *p* = 0.048); complete results for both populations are presented in Supplementary Table S2.

The maximum VIF was 1.85; VIFs were 1.21 for psychiatric history and 1.19 for previous suicide attempt. The apparent AUC was 0.710 (95% CI, 0.675–0.746). The Hosmer–Lemeshow test suggested lack of fit (χ^2^ = 20.41, df = 8; *p* = 0.009).

### 3.4. Acute In-Hospital Outcomes

ICU admission occurred in 218 patients (21.1%) and in-hospital death in 60 (5.8%). Table 4 provides separate unadjusted comparisons for these outcomes. In the adjusted ICU model, older age, psychiatric history, previous suicide attempt, higher RRRS summary score, altered mental status, and intubation were associated with ICU admission. The complete seven-covariate model is reported in Supplementary Table S1.

All 60 patients who died had altered mental status, producing quasi-complete separation in conventional mortality regression. In the Firth model, the aORs were 1.43 per RRRS summary-score point (95% CI, 1.23–1.70), 51.34 for altered mental status (95% CI, 6.69–6594.79), and 14.60 for vasopressor use (95% CI, 5.92–38.93). Psychiatric history and previous suicide attempt showed inverse adjusted associations. The wide interval for altered mental status indicates substantial imprecision, and inverse associations should not be interpreted as protective effects (Supplementary Table S1).

### 3.5. RRRS Measures and Clinical Outcomes

**Figure 2:**
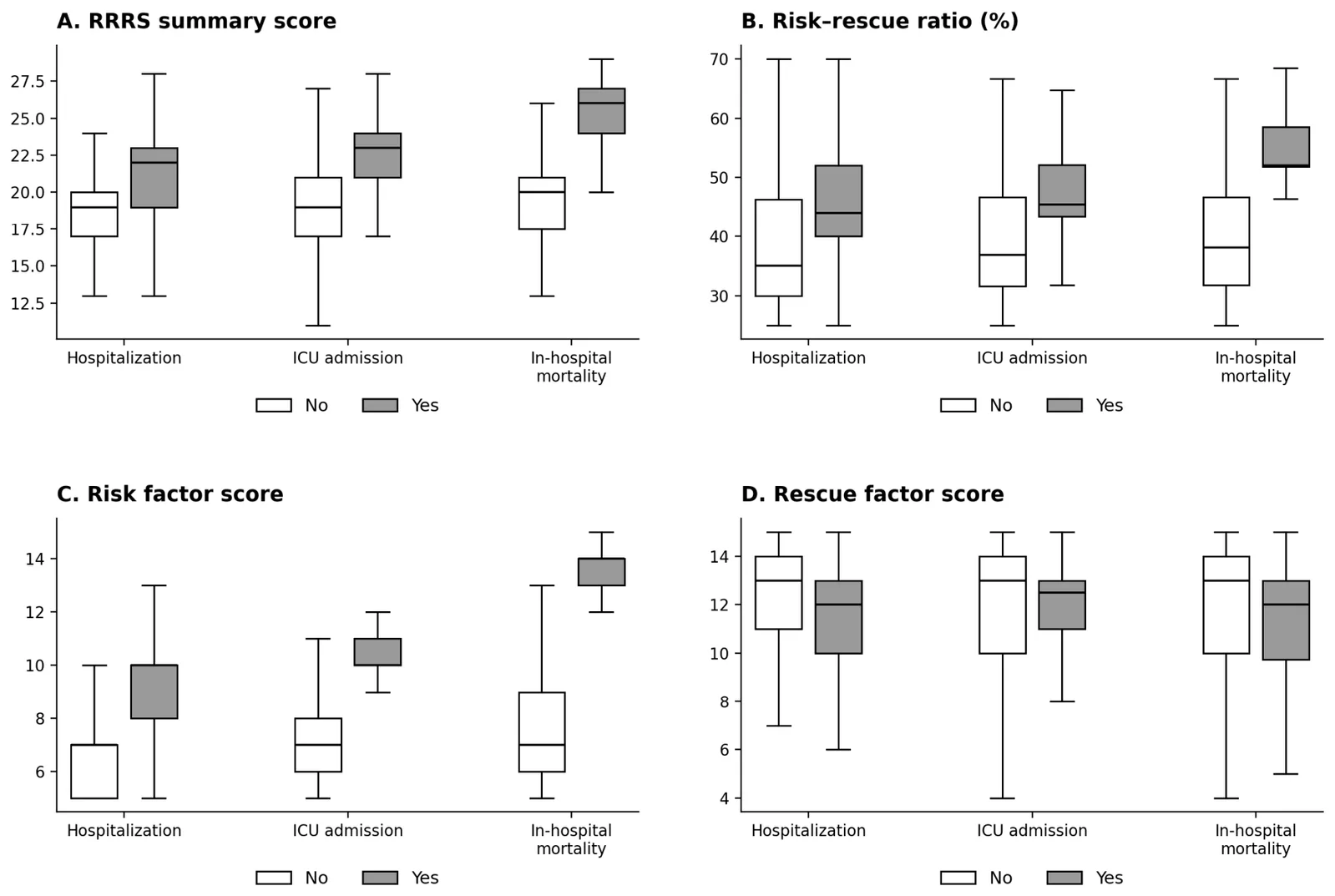
Recorded RRRS measures by hospitalization, ICU admission, and in-hospital mortality in the full cohort. (**A**), RRRS summary score; (**B**), risk–rescue ratio; (**C**), risk component; (**D**), rescue component. Boxes show IQRs, center lines medians, and whiskers the most extreme observations within 1.5 IQRs; outliers are omitted for display. White and gray boxes denote absence and presence of the outcome, respectively. These separate comparisons are not a longitudinal sequence or a validation of comparative predictive performance.

Figure 2 displays recorded RRRS measures by hospitalization, ICU admission, and in-hospital mortality. Median RRRS summary scores were 22 versus 19 for hospitalized versus nonhospitalized patients, 23 versus 19 for ICU versus non-ICU patients, and 26 versus 20 for nonsurvivors versus survivors (all *p* < 0.001). Median risk component scores were 10 versus 7, 10 versus 7, and 14 versus 7, respectively (all *p* < 0.001). Rescue scores differed for hospitalization (12 versus 13; *p* = 0.004), but not for ICU admission (12.5 versus 13; *p* = 0.241) or mortality (12 versus 13; *p* = 0.320). The risk–rescue ratio was higher in each adverse-outcome group (all *p* < 0.001). These are descriptive comparisons, not tests of comparative predictive performance.

## 4. Discussion

Among discharge survivors, 54.4% accepted hospital-based case management and 21.8% achieved successful community aftercare linkage. Linkage was confirmed in 40.2% of accepting survivors. College attendance, psychiatric history, and previous suicide attempt were associated with linkage after adjustment. The study’s contribution is the distinction between agreement to receive hospital-based support and completion of an initial transition to a community center, using feedback from the receiving service rather than referral alone. Neither outcome establishes sustained treatment participation.

The acceptance-to-linkage pathway contains several clinically and organizationally distinct steps: hospital contacts, completion of case management, referral, assessment by the receiving center, and registration. The broader literature supports coordinated psychiatric transitions and distinguishes linkage outcomes from longer-term continuity of care [14,15,16]. Qualitative evidence further shows that the experience of assessment and the perceived relevance of care matter to patients [17,18]. In our data, psychological burden, practical limitations, and low perceived need were documented among initial nonacceptors; they cannot explain the later non-linkage of accepting patients. Because intermediate completion and referral counts were unavailable, the 530-to-213 difference should not be interpreted as measured attrition at each step. Patients who did not register may also have continued care with an established psychiatrist or another provider.

College attendance may reflect differences in resources or ability to navigate services, while psychiatric history and previous attempts may indicate familiarity with care or more readily recognized follow-up needs. Korean studies of case-management completion and registration have likewise examined patient history and circumstances [28,29]. These associations support attention to access and service navigation, rather than a conclusion that education or prior suicidal behavior causes engagement. The employment estimate was close to the null boundary, and socioeconomic circumstances, health literacy, preferences, and family support were incompletely measured. Korean stigma research provides relevant context, but does not demonstrate that stigma caused non-linkage in this cohort [19]. Patients declining admission should not automatically be viewed as declining all subsequent care; our program offered follow-up regardless of DAMA status, a disposition also examined in prior ED research [30].

Endotracheal intubation had similar point estimates in the full-cohort (aOR, 0.49; 95% CI, 0.24–0.99; *p* = 0.048) and survivor-restricted (aOR, 0.52; 95% CI, 0.26–1.06; *p* = 0.073) models. The difference in nominal statistical significance is not evidence of a meaningful change in the association. The separate acute-outcome analyses describe clinical severity and treatment correlates, consistent with prior ED research on ICU admission following suicide attempts [31]. They do not identify mechanisms governing community linkage. Firth estimation addressed separation in the mortality model but did not remove the imprecision of the altered-mental-status estimate. Likewise, differences between RRRS components across outcome groups do not establish one component’s predictive superiority.

### 4.1. Limitations

Several limitations affect interpretation. This was a single-center retrospective study with a predominantly poisoning cohort. Exclusion of 61 patients with incomplete required data may introduce selection bias, and additional clinical or case-management interviews may produce differential information quality. NEDIS-based case ascertainment was not supplemented by independent adjudication of suicidal intent; cases with ambiguous documentation could not be separately characterized. Restricting the primary analysis to discharge survivors defines an appropriate post-discharge population but may induce survivor-selection bias. Registration was not modeled as a baseline exposure for in-hospital mortality, avoiding a future-linkage comparison that would require survival until linkage; however, variable follow-up opportunities and selection on survival remain relevant.

Initial registration is not a measure of attendance, care quality, or sustained engagement, and alternative care pathways were not systematically captured. Follow-up for late-2025 patients extended into 2026, but individual referral/registration dates and a common ascertainment cutoff were not available in the analysis; the outcome therefore cannot be interpreted as a fixed-time linkage rate. The records also lacked detailed patient preferences, several social determinants, community-service capacity, and re-attempts treated elsewhere. Timing of intubation and vasopressor administration relative to ICU admission was not consistently established. RRRS measures were abstracted from routine documentation rather than independently rescored. Residual confounding, exploratory multiple testing, and possible overfitting remain concerns: the events-per-coefficient ratio does not guarantee stability, and calibration showed lack of fit. The models are not validated clinical prediction tools.

### 4.2. Clinical Implications

Despite these limitations, structured clinical records and faxed outcome confirmation enabled examination of a concrete transition milestone. The findings suggest that future service evaluation should distinguish initial program acceptance, case-management completion, referral, initial linkage, and sustained engagement rather than combine them into a single measure of success. Prospective multicenter studies should record the dates and outcomes of these stages, incorporate patient perspectives and alternative care, and test whether coordinated handoffs improve longer-term outcomes, building on existing suicide-prevention implementation research [32].

## 5. Conclusions

Among 975 discharged survivors after suicide attempts, 530 accepted hospital-based case management and 213 achieved successful community aftercare linkage. College attendance, psychiatric history, and previous suicide attempt were associated with linkage. These exploratory findings identify a measurable transition between hospital support and initial community registration, without establishing causal determinants, sustained treatment engagement, or an effect on recurrent suicidal behavior.

## Figures and Tables

**Table 1:** Baseline characteristics of the full cohort (*N* = 1035).

Characteristic	Total (*N* = 1035)
Age (years)	34.0 (23.0–54.0)
Male sex	330 (31.9)
Social factors	
Employed (including students)	332 (32.1)
Living with family	501 (48.4)
Married	319 (30.8)
Religious affiliation	155 (15.0)
College attendance	144 (13.9)
Comorbidities	
Hypertension	164 (15.8)
Diabetes mellitus	88 (8.5)
Chronic kidney disease	10 (1.0)
Malignancy	30 (2.9)
Cerebrovascular disease	26 (2.5)
Psychiatric history	634 (61.3)
Attempt characteristics	
Previous suicide attempt	322 (31.1)
Time from attempt to ED arrival (h)	1.6 (0.8–5.1)
Mechanism	
Poisoning	963 (93.0)
Drowning/asphyxiation	58 (5.6)
Fall/firearm-related injury	14 (1.4)
Reported reason for attempt	
Interpersonal conflict	248 (24.0)
Work-related problems	63 (6.1)
Financial difficulties	83 (8.0)
Loss of family member	11 (1.1)
Physical illness	106 (10.2)
Unknown	524 (50.6)
Alcohol co-ingestion	261 (25.2)
Accompanied at presentation	643 (62.1)
Clinical severity	
RRRS summary score	20.0 (18.0–22.0)
Risk factor score	7.0 (6.0–9.0)
Rescue factor score	13.0 (10.0–14.0)
Risk–rescue ratio (%)	40.0 (33.3–50.0)
Altered mental status (GCS ≤ 14)	360 (34.8)
Endotracheal intubation	123 (11.9)
Vasopressor use	97 (9.4)

Values are *n* (%) or median (IQR). Percentages use the full cohort as denominator. ED, emergency department; GCS, Glasgow Coma Scale; IQR, interquartile range; RRRS, Risk–Rescue Rating Scale. The RRRS summary score is the recorded sum of risk and rescue component scores; the risk–rescue ratio is 100 × risk/(risk + rescue).

**Table 2:** Characteristics of discharge survivors according to successful community aftercare linkage (N = 975).

Characteristic	Linked (n = 213)	Not Linked (n = 762)	*p*-Value
Age (years)	26.0 (19.0–42.0)	36.0 (24.0–55.0)	<0.001
Male sex	60 (28.2)	233 (30.6)	0.498
Social factors			
Employed (including students)	109 (51.2)	220 (28.9)	<0.001
Living with family	126 (59.2)	353 (46.3)	<0.001
Married	47 (22.1)	249 (32.7)	0.003
Religious affiliation	41 (19.2)	103 (13.5)	0.037
College attendance	54 (25.4)	90 (11.8)	<0.001
Comorbidities			
Hypertension	22 (10.3)	127 (16.7)	0.023
Diabetes mellitus	17 (8.0)	61 (8.0)	0.991
Chronic kidney disease	1 (0.5)	8 (1.0)	0.693
Malignancy	2 (0.9)	25 (3.3)	0.066
Cerebrovascular disease	4 (1.9)	20 (2.6)	0.534
Psychiatric history	169 (79.3)	449 (58.9)	<0.001
Attempt characteristics			
Previous suicide attempt	109 (51.2)	209 (27.4)	<0.001
Time from attempt to ED arrival (h)	2.3 (1.0–8.0)	1.6 (0.9–5.0)	0.009
Mechanism			0.909
Poisoning	207 (97.2)	740 (97.1)	
Drowning/asphyxiation	6 (2.8)	19 (2.5)	
Fall/firearm-related injury	0 (0.0)	3 (0.4)	
Reported reason for attempt			<0.001
Interpersonal conflict	64 (30.0)	176 (23.1)	
Work-related problems	21 (9.9)	42 (5.5)	
Financial difficulties	27 (12.7)	54 (7.1)	
Loss of family member	1 (0.5)	10 (1.3)	
Physical illness	27 (12.7)	77 (10.1)	
Unknown	73 (34.3)	403 (52.9)	
Alcohol co-ingestion	46 (21.6)	207 (27.2)	0.101
Accompanied at presentation	126 (59.2)	482 (63.3)	0.275
Clinical severity			
RRRS summary score	19.0 (17.0–21.0)	20.0 (18.0–21.0)	0.225
Risk factor score	7.0 (6.0–8.0)	7.0 (6.0–9.0)	0.380
Rescue factor score	13.0 (10.0–14.0)	13.0 (10.0–14.0)	0.775
Risk–rescue ratio (%)	38.1 (31.6–47.8)	38.3 (31.8–46.2)	0.826
Altered mental status (GCS ≤ 14)	54 (25.4)	246 (32.3)	0.053
Endotracheal intubation	11 (5.2)	79 (10.4)	0.020
Vasopressor use	9 (4.2)	41 (5.4)	0.499
Hospital-based post-attempt care			
Psychiatric consultation	99 (46.5)	254 (33.3)	<0.001
Financial support	54 (25.4)	47 (6.2)	<0.001

Values are n (%) or median (IQR). Percentages are calculated within each linkage group. *p*-values are unadjusted, exploratory comparisons; omnibus *p*-values are shown for mechanism and reported reason. Unknown reason is a recorded category. ED, emergency department; GCS, Glasgow Coma Scale; IQR, interquartile range; RRRS, Risk–Rescue Rating Scale.

**Table 3:** Multivariable logistic regression for successful community aftercare linkage among discharge survivors (N = 975; 213 events).

Variable	Adjusted OR	95% CI	*p*-Value
Age (per year)	0.993	0.981–1.005	0.280
Employed (including students)	1.455	0.999–2.120	0.051
Living with family	1.272	0.881–1.835	0.199
Religious affiliation	1.332	0.863–2.057	0.196
Married	0.936	0.585–1.498	0.783
College attendance	1.692	1.124–2.548	0.012
Psychiatric history	1.694	1.126–2.548	0.011
Previous suicide attempt	1.807	1.274–2.563	<0.001
RRRS summary score (per point)	1.011	0.973–1.050	0.582
Altered mental status (GCS ≤ 14)	0.904	0.613–1.334	0.611
Endotracheal intubation	0.525	0.259–1.062	0.073
Vasopressor use	0.999	0.441–2.263	0.998

All 12 variables were entered simultaneously. Binary variables compare presence versus absence; age and RRRS are per one-unit increase. Estimates and Wald confidence intervals are shown to three decimals to avoid rounding an interval across the null value. CI, confidence interval; GCS, Glasgow Coma Scale; OR, odds ratio; RRRS, Risk–Rescue Rating Scale.

**Table 4:** Clinical characteristics by acute outcome in the full cohort: (**A**) ICU admission versus no ICU admission and (**B**) in-hospital nonsurvivors versus survivors.

(**A**)			
**Characteristic**	**ICU Admission** **(n = 218)**	**No ICU Admission** **(n = 817)**	***p*** ** -Value**
Age (years)	52.5 (32.2–72.0)	31.0 (22.0–49.0)	<0.001
Male sex	79 (36.2)	251 (30.7)	0.120
Comorbidities			
Hypertension	62 (28.4)	102 (12.5)	<0.001
Diabetes mellitus	26 (11.9)	62 (7.6)	0.041
Chronic kidney disease	4 (1.8)	6 (0.7)	0.232
Malignancy	13 (6.0)	17 (2.1)	0.002
Cerebrovascular disease	13 (6.0)	13 (1.6)	<0.001
Psychiatric history	129 (59.2)	505 (61.8)	0.478
Mechanism			0.501
Poisoning	204 (93.6)	759 (92.9)	
Prescription medication	126 (61.8)	364 (48.0)	
Pesticide	11 (5.4)	13 (1.7)	
Non-prescription medication	67 (32.8)	382 (50.3)	
Drowning/asphyxiation	13 (6.0)	45 (5.5)	
Fall/firearm-related injury	1 (0.5)	13 (1.6)	
Time from attempt to ED arrival (h)	1.4 (0.8–4.6)	1.7 (0.9–5.2)	0.193
Alcohol co-ingestion	54 (24.8)	207 (25.3)	0.864
Clinical severity			
Altered mental status (GCS ≤ 14)	180 (82.6)	180 (22.0)	<0.001
Endotracheal intubation	81 (37.2)	42 (5.1)	<0.001
Vasopressor use	56 (25.7)	41 (5.0)	<0.001
RRRS summary score	23.0 (21.0–24.0)	19.0 (17.0–21.0)	<0.001
(**B**)			
**Characteristic**	**Nonsurvivors** **(n = 60)**	**Survivors** **(n = 975)**	***p*** ** -Value**
Age (years)	50.0 (38.8–68.5)	33.0 (23.0–53.0)	<0.001
Male sex	37 (61.7)	293 (30.1)	<0.001
Comorbidities			
Hypertension	15 (25.0)	149 (15.3)	0.045
Diabetes mellitus	10 (16.7)	78 (8.0)	0.019
Chronic kidney disease	1 (1.7)	9 (0.9)	0.451
Malignancy	3 (5.0)	27 (2.8)	0.250
Cerebrovascular disease	2 (3.3)	24 (2.5)	0.659
Psychiatric history	16 (26.7)	618 (63.4)	<0.001
Mechanism			<0.001
Poisoning	16 (26.7)	947 (97.1)	
Prescription medication	4 (25.0)	486 (51.3)	
Pesticide	3 (18.8)	21 (2.2)	
Non-prescription medication	9 (56.2)	440 (46.5)	
Drowning/asphyxiation	33 (55.0)	25 (2.6)	
Fall/firearm-related injury	11 (18.3)	3 (0.3)	
Time from attempt to ED arrival (h)	0.5 (0.4–0.6)	1.7 (0.9–5.5)	<0.001
Alcohol co-ingestion	8 (13.3)	253 (25.9)	0.029
Clinical severity			
Altered mental status (GCS ≤ 14)	60 (100.0)	300 (30.8)	<0.001
Endotracheal intubation	33 (55.0)	90 (9.2)	<0.001
Vasopressor use	47 (78.3)	50 (5.1)	<0.001
RRRS summary score	26.0 (24.0–27.0)	20.0 (17.5–21.0)	<0.001

(**A**) Percentages are within each outcome group, except the indented poisoning subtypes, which use patients with poisoning as denominator (ICU: n = 204; no ICU: n = 759). The mechanism *p*-value compares the three parent categories (poisoning, drowning/asphyxiation, and fall/firearm-related injury). (**B**) Percentages are within each outcome group, except the indented poisoning subtypes, which use patients with poisoning as denominator (nonsurvivors: n = 16; survivors: n = 947). Values are n (%) or median (IQR); *p*-values are unadjusted. GCS, Glasgow Coma Scale; ICU, intensive care unit; IQR, interquartile range; RRRS, Risk–Rescue Rating Scale.

## Data Availability

The data presented in this study are not publicly available due to participant privacy and the sensitive nature of the clinical and psychiatric information. Requests for access to de-identified data may be submitted to the corresponding author for review by the principal investigator and will be considered subject to applicable institutional and ethical requirements.

## Supplementary Materials

The following supporting information can be downloaded at: https://www.mdpi.com/article/10.3390/healthcare14213712/s1. Table S1, complete acute-outcome models; Table S2, full-cohort and survivor-restricted linkage models. These are supplied in the accompanying Supplementary Material file.

## Abbreviations

aOR, adjusted odds ratio; ASSIP, Attempted Suicide Short Intervention Program; AUC, area under the receiver operating characteristic curve; CI, confidence interval; DAMA, discharge against medical advice; ED, emergency department; GCS, Glasgow Coma Scale; ICU, intensive care unit; IQR, interquartile range; NEDIS, National Emergency Department Information System; RRRS, Risk–Rescue Rating Scale; VIF, variance inflation factor.
