# Supplementary material for: Community Aftercare Linkage and Acute Clinical Outcomes Among Patients Presenting to the Emergency Department Following Suicide Attempts: A Retrospective Cohort Study
Source: Healthcare (Basel). 2026 Nov 2;14(21):3712. doi: 10.3390/healthcare14213712 (PMC13762493; doi:10.3390/healthcare14213712)
Supplement: Supplementary file 1 [file healthcare-14-03712-s001.zip › healthcare-4613084-supplementary.pdf]

Supplementary Material

Community Aftercare Linkage and Acute Clinical Outcomes Among Patients Presenting to the Emergency Department Following Suicide Attempts: A Retrospective Cohort Study

Table S1. Multivariable analyses of ICU admission and in-hospital mortality (N=1,035).

Variable	ICU admission aOR (95% CI)	p-value	In-hospital mortality aOR (95% CI)	p-value
Age (per year)	1.03 (1.02–1.05)	<0.001	0.98 (0.96–1.00)	0.081
Psychiatric history	2.07 (1.28–3.35)	0.003	0.30 (0.12–0.71)	0.006
Previous suicide attempt	3.03 (1.88–4.89)	<0.001	0.19 (0.05–0.63)	0.006
RRRS summary score (per point)	1.08 (1.02–1.14)	0.006	1.43 (1.23–1.70)	<0.001
Altered mental status (GCS ≤14)	11.71 (7.45–18.41)	<0.001	51.34 (6.69–6594.79)	<0.001
Endotracheal intubation	6.47 (3.72–11.23)	<0.001	1.24 (0.48–3.05)	0.647
Vasopressor use	1.04 (0.56–1.95)	0.897	14.60 (5.92–38.93)	<0.001

ICU admission: conventional logistic regression, 218 events. Mortality: Firth penalized logistic regression, 60 events. Each model included all seven displayed covariates simultaneously. ICU intervals and p-values use Wald inference; mortality intervals and p-values use profile penalized-likelihood inference. The very wide altered-mental-status interval reflects substantial imprecision despite finite Firth estimates. CI, confidence interval; GCS, Glasgow Coma Scale; ICU, intensive care unit; OR, odds ratio; RRRS, Risk–Rescue Rating Scale.

Table S2. Sensitivity analysis of successful community aftercare linkage by analytic population.

Variable	Full cohort (N=1,035) aOR (95% CI)	p-value	Survivors (N=975) aOR (95% CI)	p-value
Age (per year)	0.994 (0.982–1.006)	0.326	0.993 (0.981–1.005)	0.280
Employed (including students)	1.465 (1.005–2.135)	0.047	1.455 (0.999–2.120)	0.051
Living with family	1.272 (0.882–1.836)	0.198	1.272 (0.881–1.835)	0.199
Religious affiliation	1.340 (0.868–2.067)	0.187	1.332 (0.863–2.057)	0.196
Married	0.956 (0.600–1.525)	0.851	0.936 (0.585–1.498)	0.783
College attendance	1.752 (1.163–2.639)	0.007	1.692 (1.124–2.548)	0.012
Psychiatric history	1.782 (1.185–2.679)	0.006	1.694 (1.126–2.548)	0.011
Previous suicide attempt	1.859 (1.310–2.639)	<0.001	1.807 (1.274–2.563)	<0.001
RRRS summary score (per point)	1.005 (0.968–1.044)	0.778	1.011 (0.973–1.050)	0.582
Altered mental status (GCS ≤14)	0.847 (0.573–1.253)	0.406	0.904 (0.613–1.334)	0.611
Endotracheal intubation	0.489 (0.241–0.993)	0.048	0.525 (0.259–1.062)	0.073
Vasopressor use	0.658 (0.300–1.446)	0.298	0.999 (0.441–2.263)	0.998

Both models contained 213 linked patients and the same 12 covariates. The full-cohort model includes 60 in-hospital deaths coded as not linked and is retained only to assess sensitivity to cohort definition, not as a causal estimate of linkage effects. These are overlapping populations; differences in nominal statistical significance are not tests of differences between coefficients. CI, confidence interval; OR, odds ratio.
